# Selective Condensation Drives Partitioning and Sequential Secretion of Cyst Wall Proteins in Differentiating *Giardia lamblia*

**DOI:** 10.1371/journal.ppat.1000835

**Published:** 2010-04-08

**Authors:** Christian Konrad, Cornelia Spycher, Adrian B. Hehl

**Affiliations:** Institute of Parasitology, University of Zürich, Zürich, Switzerland; Washington University School of Medicine, United States of America

## Abstract

Controlled secretion of a protective extracellular matrix is required for transmission of the infective stage of a large number of protozoan and metazoan parasites. Differentiating trophozoites of the highly minimized protozoan parasite *Giardia lamblia* secrete the proteinaceous portion of the cyst wall material (CWM) consisting of three paralogous cyst wall proteins (CWP1–3) via organelles termed encystation-specific vesicles (ESVs). Phylogenetic and molecular data indicate that Diplomonads have lost a classical Golgi during reductive evolution. However, neogenesis of ESVs in encysting Giardia trophozoites transiently provides basic Golgi functions by accumulating presorted CWM exported from the ER for maturation. Based on this “minimal Golgi” hypothesis we predicted maturation of ESVs to a trans Golgi-like stage, which would manifest as a sorting event before regulated secretion of the CWM. Here we show that proteolytic processing of pro-CWP2 in maturing ESVs coincides with partitioning of CWM into two fractions, which are sorted and secreted sequentially with different kinetics. This novel sorting function leads to rapid assembly of a structurally defined outer cyst wall, followed by slow secretion of the remaining components. Using live cell microscopy we find direct evidence for condensed core formation in maturing ESVs. Core formation suggests that a mechanism controlled by phase transitions of the CWM from fluid to condensed and back likely drives CWM partitioning and makes sorting and sequential secretion possible. Blocking of CWP2 processing by a protease inhibitor leads to mis-sorting of a CWP2 reporter. Nevertheless, partitioning and sequential secretion of two portions of the CWM are unaffected in these cells. Although these cysts have a normal appearance they are not water resistant and therefore not infective. Our findings suggest that sequential assembly is a basic architectural principle of protective wall formation and requires minimal Golgi sorting functions.

## Author Summary

The protozoan *Giardia lamblia* is the leading cause for parasite-induced diarrhea with significant morbidity in humans and animals world-wide, and is transmitted by water-resistant cysts. Giardia has undergone substantial reductive evolution to a simpler organization than the last common eukaryotic ancestor, which makes it an interesting model to investigate basic cellular mechanisms. Its secretory system lacks a Golgi, but trophozoites induced to differentiate to cysts generate organelles termed encystation-specific vesicles (ESVs). Previous work shows that ESVs are most likely minimal pulsed Golgi-like compartments for exporting pre-sorted cyst wall material. We tested whether the sorting function associated with classical trans Golgi networks was also conserved in these organelles. By tracking immature and processed forms of the three cyst wall proteins during differentiation we discovered a novel sorting function which results in partitioning of ESV cargo and sequential secretion of the cyst wall material. Using live cell imaging we identified reversible formation of condensed cores as a mechanism for cargo partitioning. These observations suggest that the requirement for sequential secretion of extracellular matrix components protecting Giardia during transmission has prevented the complete secondary loss of the machinery to generate Golgi cisterna-like maturation compartments; indeed, the preserved functions have been placed under stage-specific control.

## Introduction

Infectious parasite stages transmitted to a new host via the oral route (cysts, oocysts, eggs) require a highly resistant extracellular matrix to protect them in the environment and during passage through the stomach. The diplomonad *Giardia lamblia* (syn. *G. intestinalis, G. duodenalis*) is an intestinal protozoan and a leading cause for parasite-induced diarrheal disease [1]. Trophozoites in the small intestine or in culture undergo stage-differentiation to a cyst form in response to environmental cues, e.g. changes in pH, bile and/or cholesterol concentration [2],[3]. The members of this phylum have undergone strong reductive evolution resulting in minimization or loss of cellular systems and organelles such as mitochondria, peroxisomes and the Golgi apparatus [4]–[6], but despite significant advances in phylogenetic analysis their point of divergence during evolution remains elusive [7]. Comparative genomic data suggest that the complexity of cellular organization in the last common eukaryotic ancestor with respect to compartments and membrane transport was considerable [4]. Specifically, the central organelle for maturation and sorting of excretory/secretory proteins, a classical Golgi apparatus likely with a typical stacked configuration of functionally distinct cisternae, appears to have been present in this hypothetical cell. Thus, reductive evolution is the most parsimonious, albeit still unproven, explanation for the absence of a Golgi organelle and Golgi functions in Giardia trophozoites [8].

In Giardia trophozoites, secreted proteins appear to traffic directly from the endoplasmic reticulum (ER) to the target organelle or the plasma membrane [9]. In contrast, in cells differentiating to cysts secretory cargo is delayed for many hours in specialized organelles termed encystation-specific vesicles (ESVs) which arise de novo [5],[10]. ESVs contain only presorted cyst wall material (CWM) and exclude constitutively secreted proteins even during neogenesis. The CWM rapidly polymerizes upon secretion and forms the protective cyst wall (CW) on the parasite surface at 20–24 h post induction (p.i.) of differentiation in vitro. The CWM biopolymer has a surprisingly low complexity considering its effectiveness as a biological barrier. It consists of three paralogous cyst wall proteins (CWP1–3) and simple β1–3 GalNAc homopolymer chains [11]. The glycan portion constitutes ∼60% of the CW [12], but where it is synthesized and how it is exported and incorporated into the cyst wall structure is unknown. Galactosamine synthesis from glucose and its incorporation as a polymer is mediated by pathways whose components are upregulated transcriptionally and allosterically during encystation [10], [13]–[16]. Synthesis of CWP mRNA peaks at ∼7 h p.i. and protein export from the ER to ESVs is completed after 8–10 h p.i. [17] in parasites encysting in vitro. CWPs are sorted away from constitutively secreted proteins presumably during ER export, thus ESVs contain only presorted cargo [9]. The pulsed synthesis and sorting of the CWPs to ESVs the cargo is delayed by many hours in the newly formed ESV organelle system which is best described as transient Golgi cisterna analogs [18], even though the compartments have no morphological similarity to a classical Golgi with biochemically distinct cisternae. Previously, we and others have shown transient association of COPI components with ESVs [5], ESV sensitivity to brefeldin A [9],[10], and dependence of ESV genesis and maturation on giardial Sar1 and Arf1 GTPases, respectively [18]. Taken together, there is increasing support for a model depicting ESVs as developmentally regulated, minimized Golgi-like organelles which undergo simultaneous maturation before being consumed during secretion of their cargo [8]. If confirmed, ESVs could be considered the most simply organized Golgi system identified as yet. ESVs arise stage-specifically and lack Golgi glycosyl transferases and typical structural or morphological landmarks which define this organelle in most other eukaryotes. This makes it impossible to test directly whether ESVs are indeed Golgi analogs or whether they arose independently during evolution. Thus, this issue can only be addressed by accumulation of circumstantial evidence and rigorous experimental testing of predictions based on this model.

Although few details are known it is reasonable to assume that export of the CWM is delayed in ESVs for several hours to allow for post translational maturation before it is secreted in fluid form to cover the entire cell surface where it eventually polymerizes. Proteolytic processing of CWP2 which has a 121 residue C-terminal extension rich in basic amino acids [19] is the only modification of CWPs described in any detail. Although the evidence clearly implicates a cysteine protease, there is a controversy as to which enzyme is responsible [20],[21]. In addition to processing, the enzymatic formation of disulfide [17] and isopeptide [22] bonds between CWPs appears to play a major role in the export process.

In the present study we address the question whether assembly of the giardial cyst wall requires an additional sorting step. This idea follows from a central prediction of our working model [23],[24], namely that ESVs as the only Golgi-like organelles in Giardia ultimately mature to a stage corresponding to the trans Golgi compartment of the classical Golgi whose principal function is sorting of mature cargo into distinct transport intermediates. However, a fundamental difference between ESVs and conventional Golgi cisternae is that the giardial organelles contain only a single type of pre-sorted cargo, the CWM, all of which is believed to be simultaneously secreted to the cell surface. In principle this should make a sorting step at this stage unnecessary except if the CWM were divided into distinct subfractions, for example as a result of post translational processing. This hypothesis is testable by analyzing the fate of all CWPs (pro-proteins and mature forms) by (quantitative) confocal fluorescence microscopy and Western blot. With this approach we discovered a completely novel cargo sorting function in mature ESVs resulting in partitioning of the proteinaceous CWM into two clearly defined fractions. Using specific antibodies and conditionally expressed epitope-tagged variants of CWPs we show that this processing/sorting mechanism which results in the sequential secretion of the CWM to the cell surface is necessary for the functional integrity of the cyst wall as a protective extracellular matrix.

## Results

### A dually tagged CWP2 reporter is proteolytically processed

**Figure 1:**
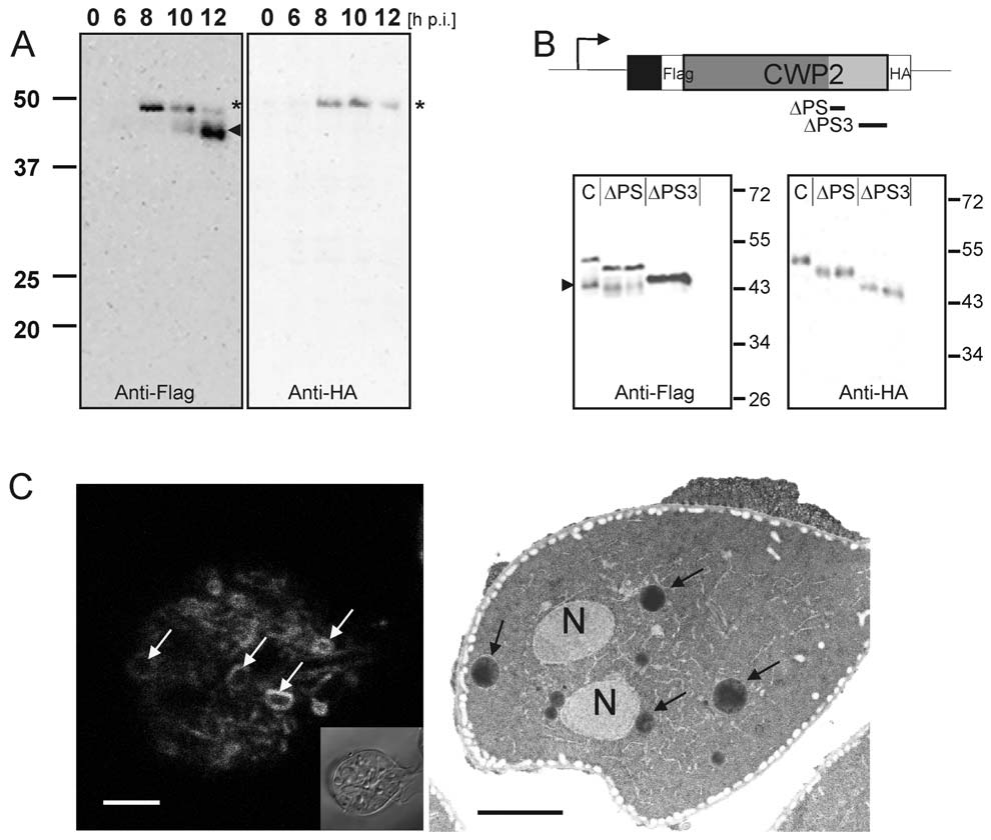
Processing of epitope-tagged CWP2 variants and CWM distribution in differentiating cells. Western blot analysis of Flag-CWP2-HA expression and processing between 0 to 12 h p.i. (A). Products (pro-form: asterisk, mature form: arrowhead) were detected in separated total lysates using anti-Flag (left panel) or anti-HA (right panel) antibodies. B) A schematic rendering of the Flag-CWP2-HA expression construct: bent arrow, CWP1 promoter; solid line, CWP1 flanking regions; boxes: signal sequence (black), epitope tags (white), CWP2 N-terminal (dark gray) and C-terminal (light gray) domains. Deleted regions in ΔPS or ΔPS3 variants are indicated. Western blots of separated crude lysates of transgenic parasites (12 h p.i.) expressing full-length Flag-CWP2-HA, or deletion products ΔPS or ΔPS3 were probed with anti-HA antibody (right panel) which labels pro-forms only while the anti-Flag antibody (left panel) labels also the large N-terminal mature forms. Note the absence of a processed form of ΔPS3. To avoid any cross-reactions two separate blots were made. C) Fluorescence and transmission EM micrographs of parasites 12 h p.i. Typical “ring-like” distribution of CWP1 in ESVs (left panel, arrows) detected with a specific monoclonal antibody. Accumulation of electron dense material in ESVs (arrows) suggests condensation of cargo (right panel, arrows). N, nucleus; scale bars 2 µm. Inset: bright field differential interference contrast (DIC) image.

Previous investigations of transport and secretion of the CWM provided evidence for proteolytic processing of the C-terminal extension of CWP2 [25]. The data suggested cleavage of the entire C-terminal extension of ∼13 kDa. However, the small C-terminal portion of the native or the transgenic CWP2 has never been visualized directly [26]. Processing of CWP2 was found to occur before secretion of the CWM but has not been correlated with expression kinetics or maturation and morphology of ESVs. To determine the temporal and spatial distribution of pro-CWP2 and its mature products we engineered a dually tagged CWP2 variant (Flag-CWP2-HA) for conditional expression under the CWP1 promoter (Figure 1A, B) [18]. Western analysis showed stage-specific expression of pro- Flag-CWP2-HA and appearance of a large processed form with a *M_R_* reduced by ∼5 kDa between 8 h and 10 h post induction (p.i.) (Figure 1A). The data are consistent with removal of a short C-terminal portion with the attached HA tag (ΔC-HA) from Flag-CWP2-HA which appears to be nearly complete at 12 h p.i. We were unable to resolve ΔC-HA on SDS-PAGE although it is readily detected in immunofluorescence microscopy analysis (IFA) (see below). To make a rough determination of the proteolytic cleavage site we expressed two modified Flag-CWP2-HA variants containing deletions from N244 to A272 (ΔPS) or A300 to V359 (ΔPS3) in the C-terminal domain (Figures 1B and S1). Western blot analysis of protein from transgenic cells at 10 h p.i. confirmed processing of ΔPS but not of ΔPS3. Combined with the apparent mass difference after removal of ΔC, this is consistent with cleavage of Flag-CWP2-HA ∼50–60 amino acids from the C-terminus.

### Post translational processing of pro-CWP2 coincides with spatial partitioning of cyst wall material

**Figure 2:**
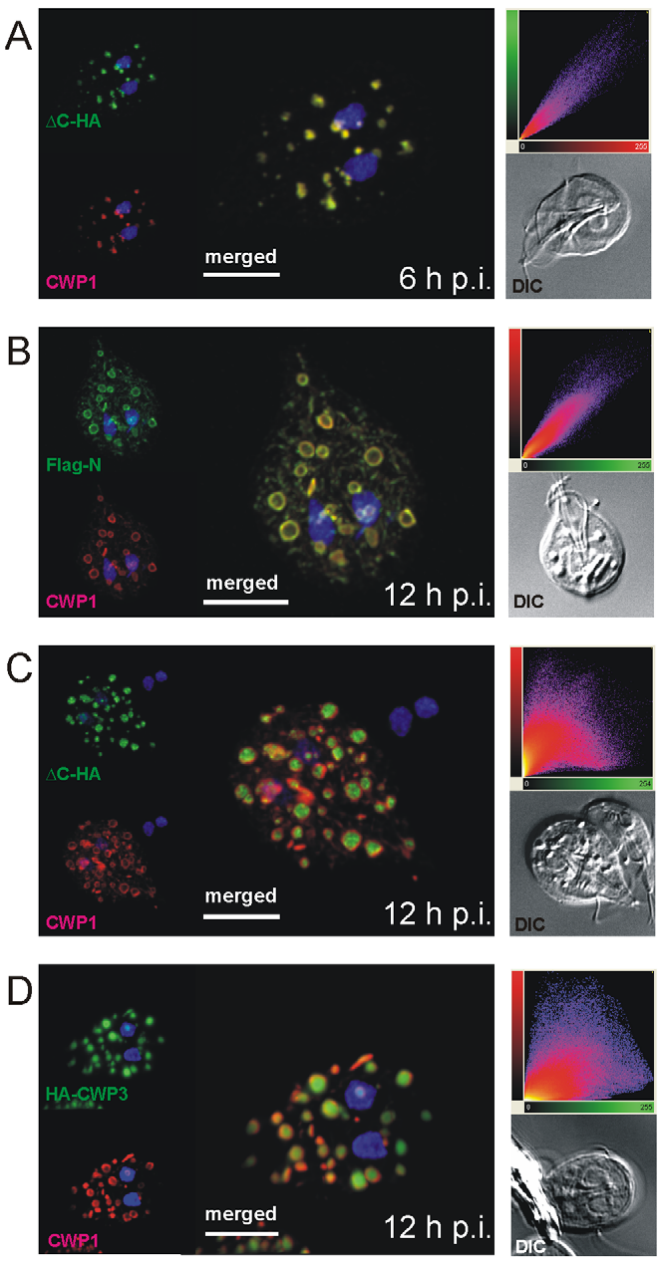
Confocal microscopy of Flag-CWP2-HA and HA-CWP3 reporters in representative cells during early stages of encystation. CWP1 (red) is used as a counter stain throughout. Insets: DIC images; scatter plots show the results of colocalization analyses of the respective markers in the entire image stacks. A) Signals from unprocessed Flag-CWP2-HA (green) overlaps with CWP1 (red) in emerging ESVs at 6 h post-induction. B, C) Partitioning of processed Flag-CWP2-HA at 12 h p.i.: The N-terminal Flag-N overlaps with CWP1 in a “ring-like” distribution, whilst the C-terminal ΔC-HA fragment is concentrated in the center of ESVs resulting in dramatically reduced overlap with CWP1. D) Like ΔC-HA, HA-tagged CWP3 (HA-CWP3) partitions in the central region of ESVs. Nuclear DNA is stained with DAPI (blue). Scale bars: 3 µm.

During the 20–24 h process of encystation, ESVs arise de novo through export of CWM from the ER and attain their final dimension of ∼500 nm between 8 h and 10 h p.i [9]. As a standard marker to follow this organelle development we and others use a commercially available mAb against CWP1. The signal observed in optical sections of ESVs generated by confocal IFA in encysting trophozoites at 10–12 h p.i. showed a characteristic ring-like distribution of the anti-CWP1 antibody (Figure 1C) compared to an even staining of organelle contents typical for earlier stages (see also Figure 2A). The ring-like distribution of the CWP1 marker was a transient phenomenon and disappeared after a few hours. The simplest explanation for this observation was that the CWM in more mature ESVs became condensed during maturation which limited penetration of the anti-CWP1 antibody into the organelle. This was also consistent with the conspicuous electron density of ESV contents (Figure 1C). However, unlike in secretory granules of other unicellular organisms, e.g., rhoptries of *Toxoplasma gondii* [27] or dense core granules of *Tetrahymena termophila* [28], no lattice-like structure is detected in transmission EM micrographs of ESVs. Together with the demonstrated exchange of a soluble CWP1::GFP reporter within an ESV organelle network [18] at this stage of the encystation process this would argue against condensation in ESVs. As an alternative explanation we therefore considered that CWP2 and/or CWP3 could be involved in the formation of a putative core which excludes CWP1. To test this we performed high-resolution confocal IFA in cells expressing the Flag-CWP2-HA reporter under stage-specific control. In differentiating transgenic cells (Figure 2) we labeled developing ESVs using the anti-CWP1 antibody (red) and detected the N- or the C-terminus of the CWP2 reporter with the anti-HA or the anti-Flag antibody, respectively (green). At 6 h p.i. the HA and the CWP1 signals overlapped completely in ESVs (Figure 2A, merged image) as documented by co-localization analysis based on the three-dimensional reconstruction of all optical sections (scatter plot). Until at least 8 h p.i. the tagged CWP2 reporter is not processed (Figure 1A). Consistent with this, the Flag and the CWP1 signals also overlapped completely in ESVs (data not shown). This was still true in cells at 12 h p.i. although both markers now showed the typical ring-like distribution of the proteins at the periphery of ESVs (Figure 2B). ΔC-HA, on the other hand, had a completely different distribution at this stage and localized inside the ring-like staining pattern of the anti-CWP1 antibody (Figure 2C). This visual assessment was confirmed by quantitative analysis of the confocal image stack which demonstrated significant loss of signal overlap (scatter plot). The same characteristic distribution was found when a CWP2 monoclonal antibody (mAb) was used in combination with anti-HA instead of the mAb against CWP1 (Figure S2). As also shown below the anti-CWP2 mAb reacts with an epitope in the N-terminal portion of CWP2. The combined data was direct evidence for the physical separation of the ΔC-HA and Flag-N products consistent with proteolytic cleavage of the pro-protein as documented in Figure 1A. Thus, the small ΔC-HA fragment was a first marker localizing to a putative core of ESVs. The observed cargo partitioning was unaffected by swapping of epitope tags on the CWP2 reporter (data not shown). To complete this analysis of CWPs in fixed cells we conditionally expressed a HA-CWP3 reporter cloned in the same vector. Analysis of tagged CWP3 by Western blot indicated that, as for the closely related CWP1, this protein was not processed by proteolytic cleavage (data not shown). Interestingly, by confocal IFA, HA-CWP3 appeared also clearly segregated from CWP1 and localized to the same central portion of ESVs as did ΔC-HA (Figure 2D). Taken together, this is direct evidence for a partitioning of the CWM inside ESVs into two separate and physically distinct fractions, each containing a CWP2-derived product.

### Does a CWM fraction form a condensed core in ESVs?

**Figure 3:**
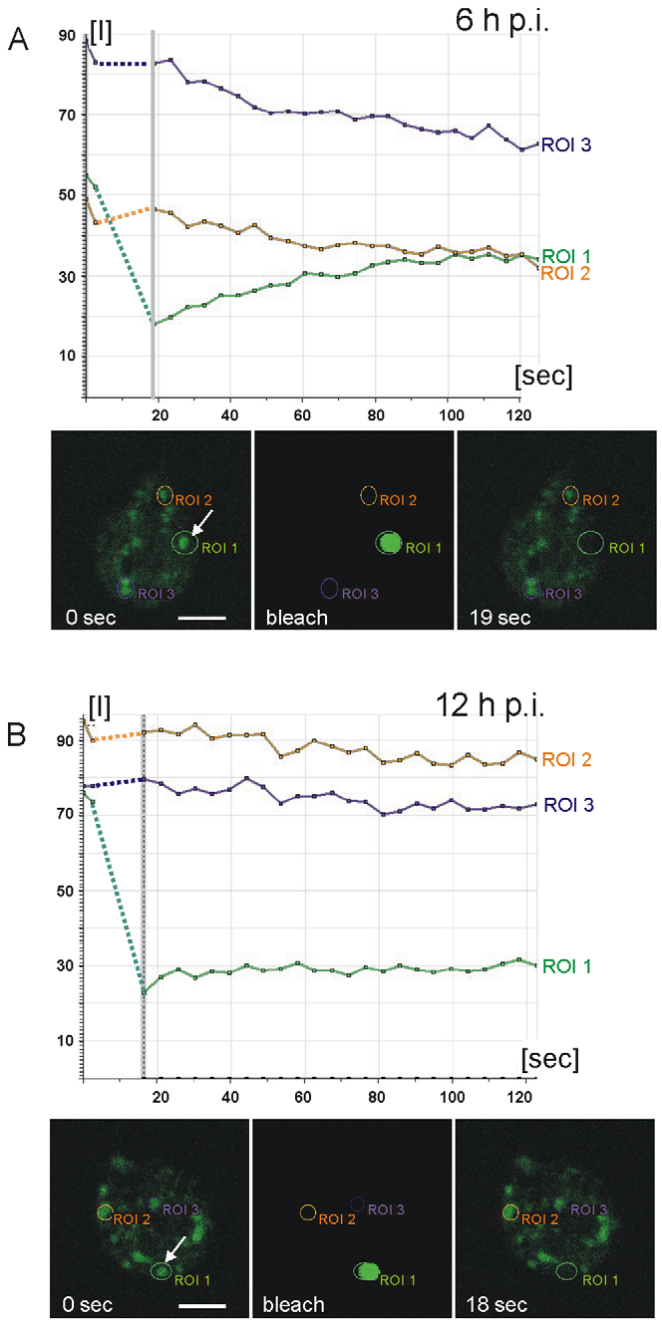
Fluorescence recovery after photobleaching (FRAP) analysis reveals condensation of CWP3::GFP during ESV maturation. Quantitative analysis of cargo motility: Photobleaching of a single ESV (region of interest 1 (ROI 1) arrow) in a living cell at 6 h p.i. (A) results in recovery (green line in graphs) of fluorescence (see also Figure S3A) with similar kinetics as a CWP1::GFP reporter [18]. Purple and amber lines represent unbleached control organelles (ROIs 2 and 3). Fluorescence micrographs from the image series at the start (0 sec) of the experiment, during bleaching, and at the beginning of the recovery phase (19 sec) are shown. In cells at 12 h p.i. (B) fluorescence in a bleached ESV (ROI 1, arrow) does not recover (see also Figure S3B) which is consistent with immobilization of CWP3::GFP in a condensed core. Arbitrary units of fluorescence are indicated [I]. Unbleached control organelles (ROIs 2 and 3) are indicated. Broken lines connect pre- and post bleaching values in the graph.

Partitioning of CWP1 from ΔC-HA within ESVs, together with the fluid nature of a CWP1::GFP reporter documented previously [18], strongly suggested that these components of the CWM assumed different physical states. Since HA-CWP3 showed the same distribution as ΔC-HA, this allowed us to directly test the hypothesis that the mechanism for cargo partitioning was indeed formation of a condensed core in ESVs. Fluorescence recovery after photobleaching (FRAP) was used to quantify the degree of mobility of a CWP3::GFP reporter in the ESV organelle system in living transgenic cells. In analogy to the experiment with CWP1::GFP [18], exchange of CWP3::GFP between ESVs was used as a measure of condensation and core formation. We tested this in cells prior to appearance of mature ESVs at 6 h p.i. and found clear evidence for recovery of fluorescence in bleached organelles (Figure 3A, quantitative analysis). Recovery showed similar kinetics as observed previously for CWP1::GFP [18], which proved that in principle the soluble CWP3::GFP reporter could be transported between ESVs. In contrast, in cells at 12 h p.i. which contained maturing ESVs, recovery of fluorescence was consistently absent (Figures 3B and S3A, B). Note also the higher rate of fluorescence loss in control organelles (6 h time point) due to dilution of the GFP pool within the ESV system during the recovery period (compare quantitative analyses in Figures 3A and 3B). To show that CWP3::GFP is immobilized in ESV cores but CWP1::GFP is not we performed fluorescence loss in photobleaching (FLIP) experiments in transgenic cells at 12 h p.i. We used six rapid cycles to bleach fluorescence in all but one ESV and quantified fluorescence loss in this organelle as a measure of diffusion in the ESV system (Figure S3C, D). Consistent with previous observations of CPW1::GFP mobility and the FRAP analysis of CWP3::GFP presented herein, we find rapid diffusion and signal loss in ESVs containing CWP1::GFP compared with the sustained fluorescence of CWP3::GFP in ESVs at this stage of encystation. Taken together, this is direct evidence for virtually complete immobilization of the CWP3 reporter in mature ESVs and consistent with core formation and loss of solubility. Combined with previously reported FRAP data using CWP1::GFP [18] this strongly supports the idea that a hallmark of maturing ESVs is partitioning of the CWM into two fractions with distinct physical properties.

### Two CWM fractions are sorted to temporally and spatially distinct secretory routes

**Figure 4:**
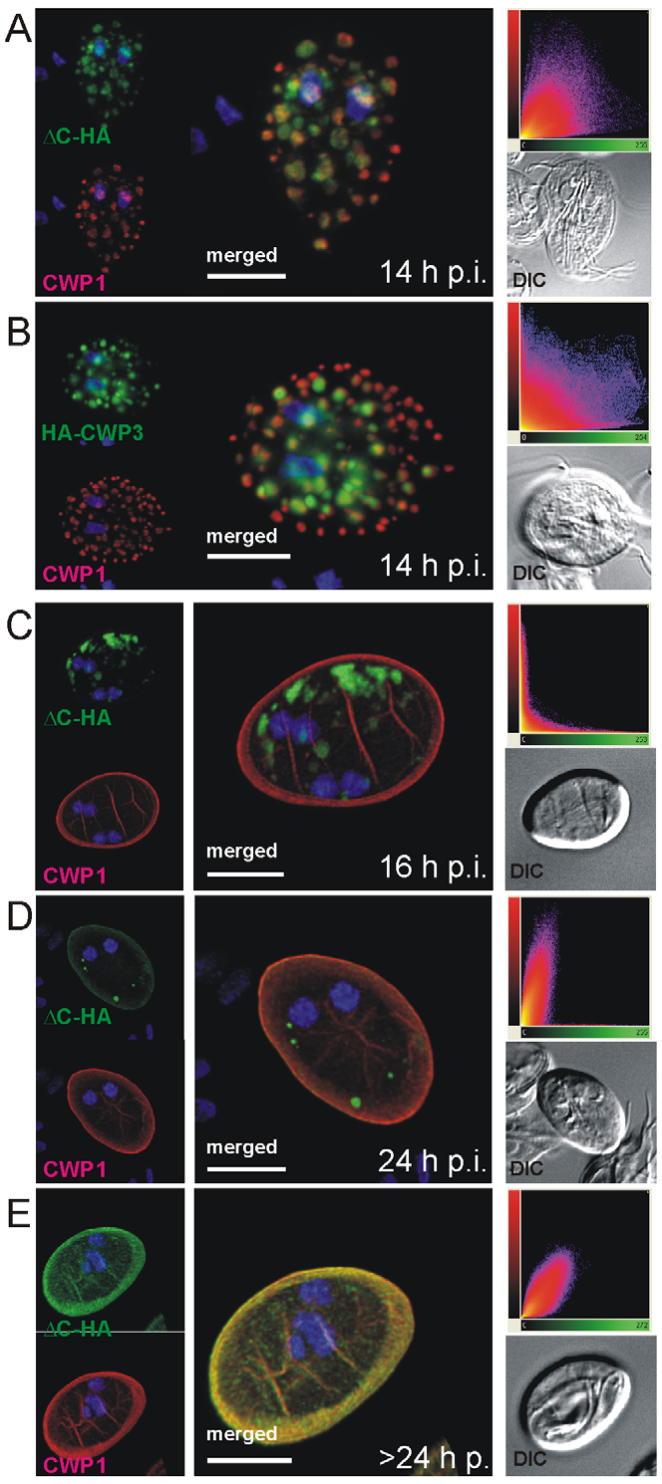
Confocal microscopy of the Flag-CWP2-HA and HA-CWP3 reporters in representative cells during late stages of encystation. In transgenic cells at 14 h post-induction, CWP1 (red) appears to be sorted away from ΔC-HA (A) or HA-CWP3 (B) to compartments at the cell periphery. In cysts emerging between 16–24 h p.i (C), CWP1 appears quantitatively secreted to the cyst wall when morphological transformation takes place whilst all ΔC-HA (green) remains in internal compartments. ΔC-HA is secreted at a later time (D, E) and eventually fully incorporated into the cyst wall. Images show representative cysts (24 and 26 h p.i., respectively) at different stages of maturation. Insets: DIC images; scatter plots show the results of colocalization analyses of the respective markers in the entire image stacks. Nuclear DNA is stained with DAPI (blue). Scale bars: 3 µm.

Sorting mechanisms based on selective condensation of secretory cargo and formation of condensed cores in the trans Golgi network (TGN) and in post Golgi vesicles of mammalian cells have been described by the “sorting by retention model” [29],[30]. In analogy, condensation of CWM components in maturing ESVs suggested that this cargo is selected for differential secretion. Indeed, in transgenic cells at 14–16 h p.i. dual labeling revealed that cargo partitioning in ESVs gave way to actual sorting of cargo into separate compartments (Figure 4A, B). The fraction consisting of CWP1 and the large N-terminal portion of the processed CWP2 (collectively termed CWMfl), presumably remained in a fluid state throughout, and appeared to be concentrated in small compartments with peripheral localization in the cell. Tagged ΔC and CWP3 proteins, collectively termed CWMco), were detected in organelles with a more central localization. Thus, whilst the mechanism for partitioning of the CWM within ESVs (i.e. physical separation of the two fractions) is clearly condensation, the cellular machinery for the subsequent sorting of CWMco and CWMfl into distinct organelles remains to be identified, but possibly involves coat protein complexes. This idea is also based on localization studies which show that clathrin (CLH) is specifically recruited to membranes of maturing ESV (Figure S4A) [8],[31]. CLH is not upregulated during encystation but the protein appears to re-localize from the membranes of the endosome-lysosome-like peripheral vesicle organelles to ESVs. CLH is most abundant on maturing ESVs with evidence for a condensed core, and appears to lose this association as sorting progresses (Figure S4B, C). Whether clathrin is directly involved in sorting of CWMfl or has another role remains to be determined. Classical clathrin coated pits on ESV membranes, at least, have never been demonstrated by electron microscopy.

The significance of this sorting event only became evident when newly formed cysts were analyzed by IFA at 16 h p.i. (Figure 4C). The CWMfl fraction (represented here by CWP1) was secreted quantitatively whereas CWMco (represented by ΔC-HA) remained in internal compartments. Correspondingly, CWMfl and CWMco lost colocalization completely as CWMfl was deposited on the outer face of the plasma membrane during morphological differentiation of the trophozoites into cysts (Figure 4C). Yet, if the cysts were allowed to mature longer and were harvested at 24 h p.i., all cysts showed partial and some even full recovery of marker colocalization at the cyst wall (Figure 4D, E) as documented in the quantitative analysis (scatter plots). This suggested that ΔC-HA, as well as CWP3-HA or CWP3::GFP (Figure S5A–C) were secreted with clearly different kinetics, suggesting a requirement for sequential deposition of the CWM fractions.

**Figure 5:**
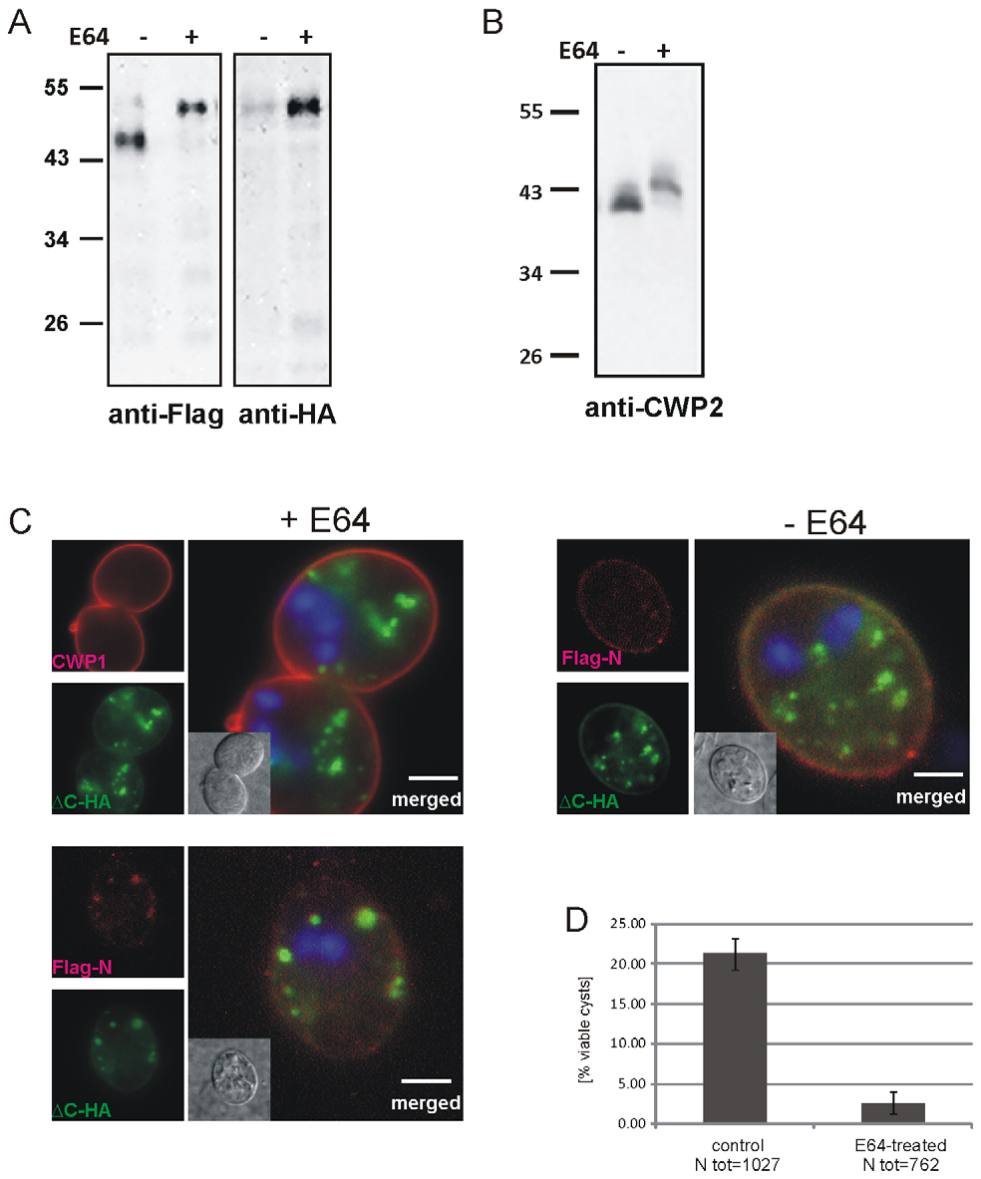
Inhibition of CWP2 and Flag-CWP2-HA processing by E64 treatment leads to mis-targeting and formation of cysts which are significantly less water resistant. (A) Western blots of separated crude lysates of transgenic parasites (12 h p.i.) expressing Flag-CWP2-HA in the presence (+) or absence (−, DMSO alone) of E64. The drug significantly abolishes processing of the Flag-CWP2-HA pro-form as well as the endogenous pro-CWP2 (B). Immunofluorescence analysis of early cysts derived from cells expressing Flag-CWP2-HA (C) shows mis-targeting of the unprocessed reporter and extensive co-localization of the Flag (red) and HA epitopes (green) in internal compartments containing CWMco after treatment with E64 (left panels). CWP1 is sorted and secreted with CWMfl, however (upper panel). In untreated control cells (right panel) the mature N-Flag fragment of the reporter is almost completely sorted away from ΔC-HA and incorporated into the CW. Nuclear DNA is stained with DAPI (blue). Scale bars: 3 µm. Cyst derived from E64-treated cells (D) have a considerably reduced survival rate compared to untreated controls.

Partitioning of CWM, core formation and processing of tagged and endogenous CWP2 all appeared to take place around 10–12 h p.i. Together with an idea presented recently by the Lujan laboratory that CWP2 coordinated export of CWM [25], the simplest explanation was that this change of physical property was triggered by the release of ΔC. To test this we inhibited processing of the Flag-CWP2-HA reporter as well as endogenous CWP2 by treating encysting cells with the protease inhibitor E64 shown to block giardial cysteine protease 2 (CP2) [20]. The Western analysis of parasites harvested at 12 h p.i. confirmed complete inhibition of processing in these conditions (Figure 5A, B). Surprisingly, using labeled anti-CWP1 antibody as a marker we found that cyst formation was not significantly impaired. More detailed analysis of fixed transgenic cells by IFA showed that in cysts derived from treated cells, unprocessed Flag-CWP2-HA remained in internal vesicles containing the CWMco fraction (Figure 5C). General cargo partitioning and sorting of the CWMfl and CWMco fractions and sequential secretion appeared to be unaffected despite the changed composition. This suggested that in treated cells CWP1 was the only family member which was exported during the formation of the first layer of the CW, followed by the components of CWMco which now included the unprocessed CWP2 (Figure 5C). Altogether, the results indicate that proteolytic cleavage of CWP2 is not necessary to induce core formation. This leaves two possibilities for the role of CWMco components: CWP3 can induce condensation alone, or alternatively, through interaction with the ΔC portion of CWP2 independent of processing. Sequestration of unprocessed Flag-CWP2-HA in the condensed core and in CWMco compartments of E64 treated cells suggests the presence of a dominant sorting signal in the short ΔC domain. An additional conclusion from these experiments was that building of the first layer of the cyst wall whose likely function is to provide structural stability to the morphologically transformed cell appeared to be independent of CWP2 processing and trafficking. Interestingly, the truncated ΔPS3 variant of the Flag-CWP2-HA reporter, which was not processed because it lacks the cleavage site, showed an identical distribution in maturing cysts derived from untreated cells (Figure S5D) as the wild type variant in cells treated with E64. This might also indicate that cleavage has to be very precise for the N-terminal part of CWP2 to be exported with CWMfl, or that cleavage and partitioning are coupled processes.

Although two CWM fractions appeared to be secreted sequentially in differentiating cells treated with E64, we suspected that the viability of these cysts was compromised. We tested water resistance as a quantifiable hallmark of correctly formed cysts by exposing mature cysts derived from E64-treated cells and from untreated controls to cold water for >24 h. Quantification of survival rates (Figure 5D) shows that the number of viable cysts from treated cells was reduced by ∼90% after exposure to water although their cyst walls remained apparently intact. This is direct evidence that correct composition of the sequentially secreted CWM, which is achieved by processing of CWP2 and by sorting of the two products in maturing ESVs, is essential for the biological activity of cysts.

## Discussion

### The formation of complex cyst walls is a universal feature

Efficient formation of water-resistant cysts of Giardia is a major contributing factor for the world-wide distribution of this extremely successful parasite. The simple organization and genetic tractability of Giardia allow for the study of basic principles of Golgi compartment neogenesis, sorting and regulated secretion in an uncluttered system [8],[32]. More importantly, by looking for universally conserved paradigms of protein trafficking and organelle organization in the minimal secretory system of Giardia we uncovered a completely unknown mechanism for cyst wall formation. The regulated secretory pathway in Giardia is established from ER-derived transport intermediates [9]. As the only Golgi-like compartments in Giardia, ESVs are exceptional since they contain only CWM and no constitutively secreted proteins [9], [23], [33]–[35]. Thus, ESVs constitute a laterally connected network of maturation compartments which is clearly distinguishable from the ER and whose synchronous maturation can be tracked during the entire 20–24 h of the differentiation process in vitro. The exported CWM has a very low complexity: Three paralogous CWPs are very likely the major proteins of the extracellular portion of the giardial cyst wall [26],[36], in addition to a simple β1–3 GalNAc homopolymer glycan [11],[12], whose manner of integration with CWPs is unknown. A cysteine-rich membrane protein (HCNCp) which may localize also to the plasma membrane of cyst forms [37] could function as a possible link between the cyst wall and the cell surface. Thus, the structural and organizational minimization in Giardia provides unique opportunities to investigate basic principles of extracellular matrix formation. Compared to Giardia cysts, environmentally resistant infectious stages of other pathogenic protozoa have more elaborate cyst walls. While the first layer of the giardial CW is secreted rapidly [8], the *Entamoeba invadens* CW is built more gradually from soluble secreted material and is anchored by a plasma membrane bound Gal/GalNAc lectin. This in turn binds to seven Jacob glycoproteins [38],[39], which cross-link chitin fibrils to establish a structural scaffold. In an elegant study, Chatterjee et al. [40] showed that construction of this structural part of the matrix, which also includes a chitinase [41],[42] involved in its remodeling, was followed by incorporation of Jessie3 proteins which provide the “mortar” that seals it. Sequential secretion of distinct CWM fractions from different secretory organelles was observed during establishment of the three distinct layers of the Eimeria oocyst wall [43],[44]. Based on these and other models we postulate that sequential assembly of multi-layered cyst walls from protein and carbohydrate is a universally conserved albeit polyphyletic trait required for full protection of infectious stages.

### The proteolytic cleavage products of CWP2 contribute to both CWM fractions

**Figure 6:**
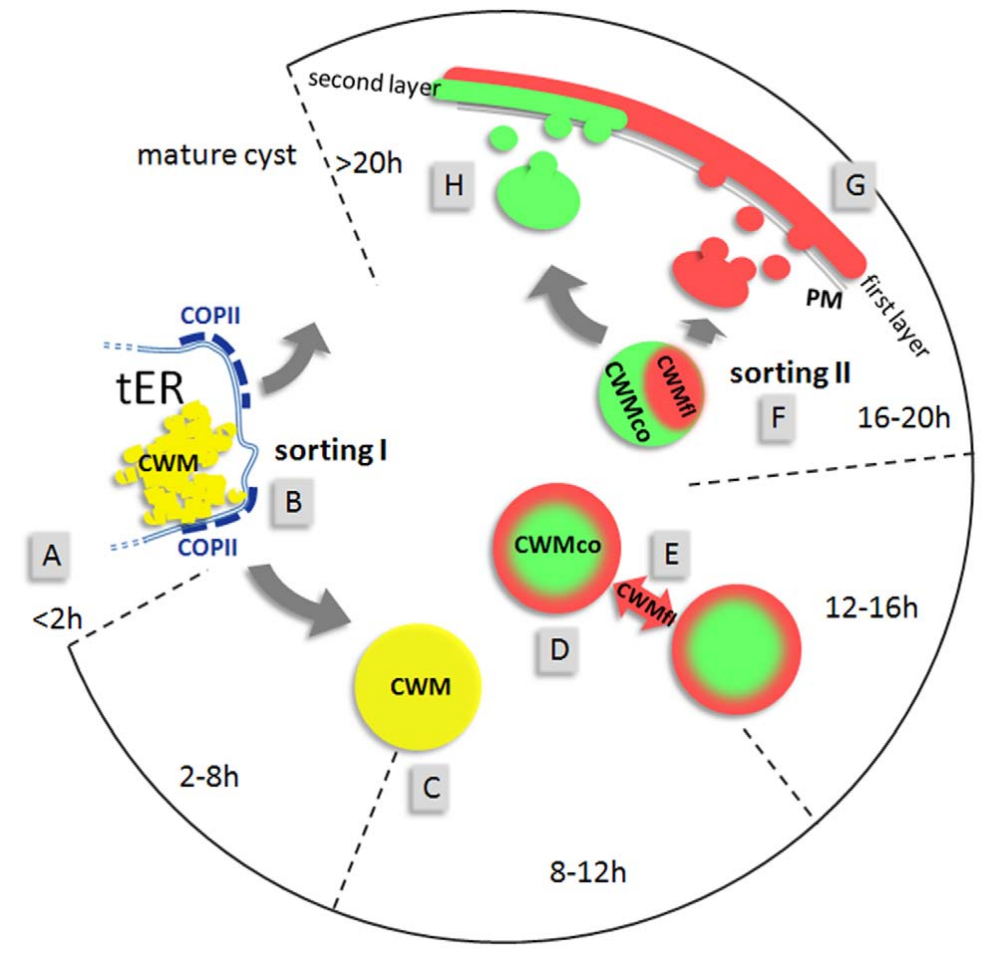
Model for the construction of the cyst wall from a single-type cargo secreted in two stages. The open circular graph depicts identifiable stages (hours post induction) of encystation. Sorting pathways are indicated by grey arrows. First appearance of CWPs in the ER (A) at about 2 h p.i. is followed by sorting of CWM from constitutively secreted cargo at transitional ER (tER) sites (B). ESVs increase in size (C) until all CWM is exported from the ER. Appearance of condensed cores in ESVs (D) coincides with processing of CWP2 and leads to partitioning of the CWM into two fractions. Maturing ESVs exchange soluble CWP1 (E) via dynamic membrane tubules. Cargo partitioning inside ESVs is followed by a sorting process separating the two CWM fractions is distinct compartments (F). CWMfl is secreted rapidly and establishes an outer cyst wall (G). Subsequent slow secretion of the CWMco fraction (H) over several hours likely requires decondensation of this material. Maturation must be completed before cysts become water resistant.

CWP2 with its prominent C-terminal extension (Figure S1) was postulated to act as an escorter for the other CWPs during export [25]. We have used a dually tagged CWP2 reporter (Flag-CWP2-HA) to investigate processing and trafficking of CWP2. In contrast to a previous report which postulated the removal of the entire C-terminal domain which is unique to CWP2 [21], our Western analysis and the examination of deletion variants indicated removal of only ∼5 kDa. Localization of C-terminally tagged CWP2 in the cyst wall by Sun and coworkers was interpreted as the presence of pro-CWP2 [26], which could mean that processing may not be required for incorporation into the matrix. Our results showed that both CWM fractions receive a portion of this domain rich in basic amino acids, indicating that it fulfills several functions. We also find evidence for the presence of a dominant sorting signal in the ΔC domain (see also below). Our data suggest that proteolytic cleavage of CWP2 is a discrete process that marks the transition from the ESV genesis to the ESVs maturation phase. Encystation is not completely synchronous in an induced population because parasites need to complete the S-G2 transition of the cell cycle in order to exit the proliferation cycle and differentiate [45]. However, our observations indicate that the transition into the maturation phase starts at ∼10 h post induction in the large majority of cells (Figure 6) and coincides with a marked downregulation of CWP synthesis [17].

Cargo partitioning provides a more sophisticated explanation for the incomplete staining by anti-CWP1 antibodies which was observed in maturing ESVs. Considering that ΔC contains a dominant targeting signal, processing can be interpreted as liberating the large N-terminal domain which remains soluble and can be secreted to the outer cyst wall layer. Since only CWP2 was processed and both its products could be detected in IFA all major players were followed either by epitope tagging or using a specific mAb in the case of CWP1 and CWP2. Investigation of cargo partitioning by high resolution confocal microscopy yields correspondingly clear cut results showing distinct localizations for these factors in either the center or the periphery of ESVs which can be quantitatively analyzed for co-localization. In addition to providing a sorting mechanism for partitioning of CWM, selective condensation theoretically allows for differential post-translational modification of components in fluid and condensed fractions. This could partially offset the lack of a stacked cisternal organization of this organelle system.

### Condensed core formation drives CWM partitioning and is a prerequisite for cargo sorting

Biogenesis of secretory granules is still poorly understood [30]. Formation of immature granules occurs at the TGN in endocrine, exocrine and neuronal cells by sorting granule proteins from constitutively secreted cargo. Condensation of soluble proteins is organized by aggregation factors such as chromogranin A of neuroendocrine cells which drive granule formation independently of coat protein complexes [46],[47]. Core formation in secretory granule biogenesis is dependent on inherent biophysical properties of cargo components and aided by acidic pH and high Ca2+ in these organelles. Our attempts to disrupt or delay this process in ESVs by inhibiting acidification of organelles using ammonium chloride or the H^+-^ATPase inhibitor bafilomycin, or by depleting intracellular calcium were not successful (Konrad and Hehl, unpublished). This suggests that an inherent tendency to aggregate is the dominant driving force of CWMco condensation. Alternatively, interaction with an as yet unidentified component could prevent circulating CWMfl components from becoming condensed. Further maturation of secretory granules in higher eukaryotes entails sorting and removal of non-granule proteins by vesicular traffic involving AP1/clathrin [48]. We have observed important recruitment of clathrin to membranes of mature ESVs (Figure S4A) [9] but this alone does not prove any involvement in the sorting of CWMfl. Furthermore, because over-expression of a clathrin hub fragment during encystation had no effect on cyst formation (Stefanic and Hehl, unpublished data) the role of this coat protein remains to be determined. More interestingly, expression of a dominant-negative Arf1 homolog which also recruits AP1/clathrin prevented secretion of CWP1 from ESVs but not morphological transformation which suggests an essential function in late steps of regulated secretion [18].

One of the principal questions in connection with ESV maturation was whether a condensed core was formed. We were able to address this directly using a CWP3::GFP reporter to compare the physical state of CWP3 as a marker for the core with that of the closely related CWP1 protein whose dynamics was investigated previously [18]. FRAP and FLIP experiments provided the key piece of evidence for the interpretation of the results obtained by fluorescence microscopy which revealed progression from cargo partitioning to sorting and sequential secretion.

E64-inhibitable proteolytic processing of pro-CWP2 has been described previously by Touz and coworkers [21] using an antibody which binds to its N-terminal portion. In agreement with these findings we detected an initial retention of unprocessed CWP2 in internal compartments. In contrast, by using an extended experimental approach, i.e., co-labeling of markers for both CWM fractions, we documented the sequential nature of CWM secretion. Interestingly, our data showed that cyst formation was completed even when processing of endogenous and recombinant CWP2 was blocked (see Figure 5). Treatment of encysting cells with E64 did not affect stage-differentiation, secretion of CWM, or formation of an extracellular matrix, even though pro-CWP2 was retained in the CWMco fraction. Retention of ΔPS3, which differs from the mature N-terminal CWP2 fragment by only few amino acids, points to a surprisingly stringent dependence on precise cleavage of pro-CWP2 or on cleavage itself, which contrasts with the overall robustness of the sorting process. Detailed investigation of this step, including identification of the proteolytic cleavage site, will be necessary to resolve the link between processing and sorting of the two pro-CWP2 products.

### An integrated model for regulated secretion in encysting Giardia

Together with results from previous investigations the sorting data presented herein provide a novel scenario for regulated export of CWM in Giardia (Figure 6). The complete pathway requires two discrete sorting steps which are both consistent with our Golgi model for ESVs: I) Sorting of CWM from constitutively secreted proteins at ER exit sites [9], and concomitant export of CWPs to ESVs. II) Partitioning and sorting of the mature CWM into two fractions shortly before secretion. Processing of CWP2 coincides with, but is not required for, condensed core formation in ESVs. The subsequent separation and sequential secretion of the physically distinct CWMfl and CWMco fractions is consistent with maturation of ESVs to TGN analogs and a “sorting by retention” mechanism for separating differentially secreted cargo [29]. In addition to being a prerequisite for subsequent sorting, condensation of CWMco may serve to sequester this soluble content cargo from modifying factors. CWMfl components, however, continue to circulate and could theoretically intersect with other compartments such as the ER or PVs. Secretion of CWMfl is completed in only a few minutes simultaneously with loss and/or resorption of flagella, disassembly of the ventral disk and nuclear division [8],[49], and most likely provides primarily structural stability to the differentiated cell. Unlike reported previously [50], we find that encysting trophozoites adhere quite well in vitro, until just prior to secretion of the CWM (Trepp, Spycher and Hehl, unpublished) when they lose attachment as the cytoskeleton is reorganized. This allows the newly formed cyst walls to reach full function before cysts are finally shed into the environment.

### Incorporation of carbohydrate into the cyst wall – the missing piece of the puzzle

Integration of chitin with early and late secreted proteins in encysting *E. invadens* is essential for establishing a fully functional cyst wall [40]. How the unique β1–3 GalNAc homopolymer chains which provide the bulk of the CW carbohydrate are integrated into this structure during encystation in Giardia remains unknown. Three factors, i.e., the fibrillar nature of the polymerized CWM in the outer cyst wall as shown in scanning EM [11], studies showing that this material is composed of carbohydrate and protein [15],[51], and the absence of specialized vesicles containing large amounts of this carbohydrate, raise the question how this material is exported. This still awaits resolution, mainly because no known lectin reacts with the carbohydrate portion of the giardial CW with sufficient specificity. The *Phaseolus lunatus* lectin (LBA) has been reported to bind to the *G. muris* CW [11], but reactivity with the *G. lamblia* CW is poor and inconsistent. More importantly, fluorochrome-conjugated LBA weakly labeled the nuclear envelope but not ESVs or other large organelles in encysting cells which might be involved in export of CW carbohydrate (Hehl, unpublished). Whatever the route of carbohydrate export, evidence for extensive covalent cross-linking [12],[24],[52] of CWPs and carbohydrate chains underscore the importance of a structurally resistant CW.

The simplest explanation for the sequential secretion of CWM components is that the fibrillar shell of polymerized CWMfl requires sealing to become fully protective and infectious. Based on the high proportion of intermediate stages (Figure 4D) found in cyst preparations at 16–24 h p.i., export of the CWMco fraction appears to be considerably slower than secretion of CWMfl, most likely because the former must be decondensed for secretion. In light of the low complexity of the CWM, investigation of the biochemistry of its reversible condensation and subsequent polymerization is expected to reveal fundamental aspects of biopolymer export and assembly. CWPs and their inherent tendency to aggregate may be the primary driving force for cargo partitioning and ultimately for polymerization on the surface. It is likely that carbohydrates play a much more important role in coordinating sequential secretion than merely providing a means for cross-linking the protein components of the CW.

## Materials and Methods

### Giardia cell culture, transfection and in vitro encystations

Trophozoites of the Giardia lamblia strain WBC6 (ATCC catalog number 50803) were grown under anaerobic conditions in 11 ml culture tubes (Nunc, Roskilde, Denmark) containing TYI-S-33 medium supplemented with 10% adult bovine serum and bovine bile according to standard protocols [17]. For chemical fixation or protein extraction parasites were harvested by chilling the culture tubes on ice for 30 minutes to detach adherent cells, and collected by centrifugation at 1000×*g* for 10 minutes. Cells were then resuspended in phosphate-buffered saline (PBS) and counted.

Encystation was induced using the two-step method as described previously [17], by cultivating the cells for 44 hours in medium without bile and subsequently in medium with porcine bile and a pH of 7.85.

Circular plasmid DNA of expression vectors was linearized at the *Swa*I restriction site [18] and 15 µg of cut DNA were electroporated into 5·10^6^ freshly harvested trophozoites on ice using the following settings: 350 V, 960 µF, 800Ω. Linearized plasmids were targeted to the *Giardia lamblia* triose phosphate isomerase (Gl-TPI) locus (see below) and integration occurred by homologous recombination under selective pressure of the antibiotic puromycin (Sigma, St. Louis, MO) for 5 days. Transgenic cell lines were maintained and analyzed without antibiotic.

### Expression constructs

For the inducible expression of tagged proteins in *Giardia*, a previously described vector pPacV-Integ was used which allows for the expression of fusion proteins with a N-terminal HA-tag under the control of the CWP1 promoter [18]. For the expression of double-tagged CWP2 (Flag-CWP2-HA), a Flag-tag was fused downstream of the stretch coding for the CWP1 signal peptide using oligonucleotide primers 44 and 768 (Table S1) to PCR amplify the CWP1 promoter including the CWP1 signal peptide from genomic DNA. The PCR product was ligated into the XbaI and NsiI sites upstream of the CWP2 coding sequence. The CWP2 open reading frame (ORF) without the stretch coding for the signal sequence (E21 - R362) was PCR amplified using oligonucleotide primers 760 and 756. The latter included the sequence coding for the HA epitope tag. This fragment was ligated in frame using the NsiI and PacI sites of the pPacV-Integ expression cassette to generate the basic Flag-CWP2-HA vector. All constructs were sequenced prior to transfection.

CWP2 deletion constructs: To express CWP2 lacking N244–A272 (ΔPS), two DNA fragments (coding for E21–R243 and H273–R362) were amplified with oligonucleotides 760 and 842, and 844 and 756, respectively, and ligated via the introduced EcoRI site, and used to replace the original NsiI - PacI fragment in the Flag-CWP2-HA vector. The same strategy was used to generate ΔPS3 lacking A300–V359 of the CWP2 ORF: the sequence coding for the E21–T299 fragment of the CWP2 ORF was PCR amplified using primers 760 and 877 and used to replace the NsiI - PacI fragment in the Flag-CWP2-HA vector.

CWP3 constructs: the CWP3::GFP expression construct was made by replacing the CWP1 sequence in a previously used construct CWP1::GFP [18] with the CWP3 ORF and promoter region PCR amplified with primers 936 and 937. An HA-tagged CWP3 (HA-CWP3) fragment was made by PCR amplifying the region coding for M17-R247 of the CWP3 ORF using primers 856 and 420 and replacing the NsiI-Pac fragment in the pPacV-Integ expression vector.

### Treatment with the cysteine protease inhibitor E64

Encystation of trophozoites was induced in the presence of 30 µM E64 or an equal volume of the solvent (control). To determine the number of viable cysts in preparations cells were harvested after 48 h, washed with PBS and incubated in ddH2O for >48 hours at 4°C. For the quantification of cyst viability cells were stained with a mixture of acridin orange (4 ug/ml) and ethidium bromide (0.1 mg/ml) in PBS for 10 min at room temperature and washed once in PBS. Cells were mounted on a slide and imaged on a Leica DM-IRBE microscope using a 40× lens (Leica Microsystems GmbH, Wetzlar, Germany). For each condition two sets of 13 randomly selected fields were digitally recorded (Diagnostic Instruments Inc., USA) and processed with the Metaview software package (Visitron Systems GmbH, Puchheim, Germany). Percentage values of replicates were averaged.

### Protein analysis

For the preparation of total cell extracts Giardia parasites were harvested as described above. The cell pellet was dissolved in SDS sample buffer to obtain of 2·10^5^ cells in 50 µl and boiled for 3 minutes. Dithiothreitol (DTT) was added to a final concentration of 7.75 µg/ml before boiling. SDS-PAGE on 12% polyacrylamide gels and transfer to nitrocellulose membranes was done according to standard techniques. Nitrocellulose membranes were blocked in 5% dry milk/0.05% TWEEN-20/PBS and incubated with the primary antibodies (anti-HA, anti-Flag, anti CWP2 mAb) at the appropriate dilution in blocking solution. Bound antibodies were detected with horseradish peroxidase-conjugated goat anti-mouse IgG (Bio-Rad, Hercules, CA) and developed using Western Lightning Chemiluminescence Reagent (PerkinElmer Life Sciences, Boston, MA, USA). Data collection was done in a MultiImage Light Cabinet with AlphaEase FC software (Alpha Innotech, San Leonardo, CA, USA) using the appropriate settings.

### Cell imaging techniques

Immunofluorescence analysis: Chemical fixation and preparation for fluorescence microscopy was performed as described [9]. Briefly, cells were washed with cold PBS after harvesting and fixed with 3% formaldehyde in PBS for 40 min at 20°C, followed by a 5 min incubation with 0.1 M glycine in PBS. Cells were permeabilized with 0.2% triton X-100 in PBS for 20 min at room temperature and blocked overnight in 2% BSA in PBS. Incubations of all antibodies were done in 2% BSA/0.2% Triton X-100 in PBS. Cells were incubated with directly coupled mouse monoclonal antibodies, i.e., Alexa488-conjugated anti-HA (Roche Diagnostics GmbH, Manheim, Germany; dilution 1∶30), Cy3 conjugated anti-Flag (Sigma, St. Louis, MO 1∶30), or Texas Red-conjugated anti-CWP1 (Waterborne™, Inc., New Orleans, LA, USA; dilution 1∶80) for 1 h at 4°C. CLH was detected with a Giardia-specific polyclonal antibody [31]. Post incubation washes were done with 0.5% BSA/0.05% triton X-100 in PBS. Labeled cells were embedded for microscopy with Vectashield (Vector Laboratories, Inc., Burlingame, CA, USA) containing the DNA intercalating agent 4′-6-Diamidino-2-phenylindole (DAPI) for detection of nuclear DNA. Immunofluorescence analysis was performed on a Leica SP2 AOBS confocal laser-scanning microscope (Leica Microsystems, Wetzlar, Germany) equipped with a glycerol objective (Leica, HCX PL APO CS 63× 1.3 Corr). Confocal image stacks were recorded with a pinhole setting of Airy 1 and twofold oversampling. Further processing was done using the Huygens deconvolution software package version 2.7 (Scientific Volume Imaging, Hilversum, NL). Three-dimensional reconstructions and quantitative analysis of co-localization were done with the Imaris software suite (Bitplane, Zurich, Switzerland). Alternatively, a standard fluorescence microscope (Leica DM IRBE) and MetaVue software (version: 5.0r1) was used for data collection.

Live cell microscopy, fluorescence recovery after photobleaching (FRAP) and fluorescence loss in photobleaching (FLIP) analysis: For live cell microscopy, induced cells expressing the CWP3::GFP chimera were harvested at 6 or 12 h p.i. and transferred to 24-well plates at a density of 6·10^6^/ml. After incubation on ice for 5–8 h, oxygenated cells were sealed between microscopy glass slides and warmed to 21°C or 37°C. Under these conditions, the encysting cells were stable and even continued to complete encystation. For FRAP, FLIP and time-lapse series, images were collected on a Leica SP2 AOBS confocal laser-scanning microscope (Leica Microsystems, Wetzlar, Germany) using a 63× water immersion objective (Leica, HCX PL APO CS 63× 1.2 W Corr). Fluorescence in selected regions of interest was quantified using the corresponding Leica software suite. The pinhole was set to Airy 2 in order to increase the thickness of the optical sections to accommodate an entire ESV in the z-plane. Quantifiable criteria for cell viability were active attachment to substrate and continuous beating of the ventral and anterolateral flagella pairs. FRAP experiments were performed with the same settings as used for the CWP1::GFP reporter [18] with Leica FRAP software module to set bleaching parameters and to quantify fluorescence recovery.

Electron microscopy: Encysting parasites were prepared for TEM as described previously [18]. To achieve uniform orientation, ultrathin sections were cut parallel to the sapphire surface, stained with uranyl acetate and lead citrate and examined in a CM12 electron microscope (Philips) equipped with a slow-scan CCD camera (Gatan, Pleasanton, CA, USA) at an acceleration voltage of 100 kV. Recorded pictures were processed further with the Digital Micrograph 3.34 software (Gatan, Pleasanton, CA, USA).

### Accession numbers

GiardiaDB accession numbers are given for the following genes: CWP1 GL50803_5638, CWP2 GL50803_5435, CWP3 GL50803_2421, clathrin heavy chain (CLH) GL50803_102108.

## Supporting Information

Table S1 Sequences of oligonucleotide primers used for PCR amplification.
(0.04 MB PDF)

Figure S1 Amino acid sequence alignment of CWP1–3 generated with CLUSTAL W. Identities are indicated with an asterisk, similar amino acids with dots. The localization of the deletions in the CWP2 variants ΔPS and ΔPS3 is indicated.
(0.01 MB PDF)

Figure S2 Localization of endogenous CWP2. Giardial CWP2 (red) and ΔC-HA (green) in a representative encysting cell (A) and in maturing cysts (B–D). An anti-CWP2 mAb was used to confirm the localization of the N-terminal processing product of CWP2 in differentiating cells between 12 h and >24 h p.i. Cells at 12 h p.i. show clear evidence for cargo partitioning (arrows) and sorting (arrowheads). Scale bar 5 µm.
(0.20 MB PDF)

Figure S3 FRAP and FLIP analysis. Single frames from the FRAP analysis of the CWP3::GFP reporter (A, B). Pre bleach, post bleach and endpoint (120 sec) of the FRAP time-lapse series (28 images each) shown in Figure 3. First Row (A) 6 h p.i., second row (B) 12 h p.i. Arrows point to bleached organelle. Fluorescence loss in photobleaching (FLIP) experiments with CWP1::GFP (C) and CWP3::GFP (D) in cells at 12 h p.i. All GFP fluorescence in ESVs except in a single target organelle (ROI 1, arrow) was photobleached with 6 rapid cycles. The mobility of the remaining fluorescent reporter was quantified over 120 sec (graphs). Almost complete loss of CWP1::GFP fluorescence in the target organelle is consistent with mobility of the reporter in the ESV system. Conversely, the CWP3::GFP signal remains intact, consistent with immobilization due to incorporation into a condensed structure within ESVs.
(0.43 MB PDF)

Figure S4 High resolution confocal microscopy of clathrin and secreted CWM. Clathrin heavy chain (CLH) is recruited to ESVs in cells showing evidence for cargo partitioning. CLH (green or blue) is detected by a specific antibody against the giardial protein [31] and the anti-CWP1 mAb (red) and/or anti-HA (green) is used to localize CWMfl and CWMco, respectively. Dual labeling (A) of encysting cells at 12 h p.i. showing full cargo partitioning (I) and beginning sorting (II and III) of CWP1. Recruitment of CLH to ESVs is most pronounced before and also during sorting. Three dimensional reconstructions of deconvolved optical sections and quantification of colocalization is shown (scatter plot; signal in boxed area represents CLH localized to peripheral vesicles). Triple labeling (B and C) of cells at 12 h p.i. showing full cargo partitioning (B) and sorting (C) of CWP1 (red) and ΔC-HA (green). CLH (blue) distributes to endosomal-lysosomal peripheral vesicles as shown previously [31], and is recruited to ESV membranes (arrows and arrowheads). Single optical sections (top rows) and three dimensional reconstructions (bottom rows) are shown. Scale bars 5 µm.
(0.20 MB PDF)

Figure S5 Localization of tagged CWP3 variants in cysts. Confocal fluorescence microscopy images of HA-CWP3 at early (A) and late (B) stages of cyst maturation. CWP3::GFP shows identical distributions in maturing live cysts (C). Scale bar 5 µm. (D) Localization of the uncleavable ΔPS3 variant of CWP2 shows the typical distribution of material exported with the CWMco fraction in maturing cysts. Scale bar 5 µm.
(0.09 MB PDF)
