# Supplementary material for: Selective Condensation Drives Partitioning and Sequential Secretion of Cyst Wall Proteins in Differentiating Giardia lamblia
Source: PLoS Pathog. 2010 Apr 8;6(4):e1000835. doi: 10.1371/journal.ppat.1000835 (PMC2851657; doi:10.1371/journal.ppat.1000835)
Supplement: Table S1 — Sequences of oligonucleotide primers used for PCR amplification. (0.04 MB PDF) [file ppat.1000835.s001.pdf]

Oligonucleotide primers (5'- 3' orientation) used in this study:

Nr.	Sequence
44	ATTCTAGACTAGCCACGCATGGGCTGT
420	CCTTAATTAATTATCTGTAGTAGGGCGGCTGTATC
756	CGTTAATTAATCACCTTCTGCGAGCGTAGTCTGGGACATCGTATGGGTAAGCG ACAATAGGCTTGTTCGTGCTG
760	GCATGCATGAGGAGGAGGCCCTGACCAATTTG
768	GCATGCATCTTGTCATCGTCGTCCTTGTAGTCGACGAGCACCTCCCTCTGA
842	GAGAATTCGCGGACTACTGTCTGCCTG
844	GAGAATTCGCCTCCAACTGTGGCAAG
856	GACCTGCAGGACATGCAATATGATGCACTCGTTCAGTTTTATG
877	CGTTAATTAATCACCTTCTGCGAGCGTAGTCTGGGACATCGTATGGGTACGTCT TGCTAGATGACCTAACCA
936	GAGCTAGCGAGATAGGAGAATACTACCTG
937	GACCTGCAGGTATCTGTAGTAGGGCGGCTG
