# Supplementary material for: Selective Condensation Drives Partitioning and Sequential Secretion of Cyst Wall Proteins in Differentiating Giardia lamblia
Source: PLoS Pathog. 2010 Apr 8;6(4):e1000835. doi: 10.1371/journal.ppat.1000835 (PMC2851657; doi:10.1371/journal.ppat.1000835)
Supplement: Figure S1 — Amino acid sequence alignment of CWP1–3 generated with CLUSTAL W. Identities are indicated with an asterisk, similar amino acids with dots. The localization of the deletions in the CWP2 variants ΔPS and ΔPS3 is indicated. (0.01 MB PDF) [file ppat.1000835.s002.pdf]

```
CWP1 MMLALLALAGSALA-LTCPATQREVLVEIYDATDGANWKTNNWLSGD-SICTWTGVTCEA 58
CWP2 -MIAALVLGLLGLARAACPATEEEALTNLYDALDGANWKSNNWLTPDVSYCSWTGITCDS 59
CWP3 -MFSLLLLLEVGYG---LVDMQYDALVQFYDSTDGANWMPNNWLQSD-VYCDWIGVSCDD 55
      *:: * *   . .       : :.*.::**: ***** .****  *   * * *::*:

CWP1 SNNYVIALDLSDMGLTGTIPENIGCLTYLKTLYLSNNSLAGAIPEGLCQLTNLQYLQVNS 118
CWP2 NNN-VIGIDLSDMGLTGALPADIGCFPLLRSLYLNNNDLAGPIPTDLCALTSMQYLQINN 118
CWP3 NDN-VVSLNVQHMGLNGQL-SDLTNLTYLSSLYLSGNNLKDSDLCLLGGLSYLRVLDMTD 113
     .:* *:.:::..***.* :  ::  :. * :***..*.* ..    *  *: :: *::..

CWP1 AGLTGDIPECMCDLIHLMFWYMSDNALTGSIPTCINELQFLKELHLDCNQLSGTVPVGLM 178
CWP2 AGLTGDIPECICDLTHMMFWYMSINALTGPIPTCVNELQFLKELHLDCNELTGDVPADLF 178
CWP3 TSLDGNIPECICALSKLHSLHLDNNSLIGDVPPCLGDSQQLGLKLFTARCNRLQYSFSHL 173
     :.* *:****:* * ::   ::. *:* * :*.*:.: * *    : ..      . . :

CWP1 TLPYLMELYLNCNPDLTCP--DATGVQF-VFKCGDVDCENCGTLPPTNCAQCFTDPDCGE 235
CWP2 DLPYMMEIHVQCNTDLVCT--AAPDTYTGIYLCGTTDCDYCTALPPTNCPTTLERDGCTY 236
CWP3 DLAVVDYVDLQCNPTINCGGEDYVVNHTGYYACGLNHCSTC--VKKTTCAAFLDVGGCRY 231
      *. :  : ::**. : *            : **  .*. *  :  *.*.  :   .*

CWP1 YCLTPP------------------------------------------------------ 241
CWP2 YRQTVVRNASGRKTSCNARSASNCGKAKSNMHNSAHNAQRKCNMPNSRSQTPLRTVVRSS 296
CWP3 YLRNSTASKIQPPYYR-------------------------------------------- 247
     * .                 ΔPS

CWP1 ------------------------------------------------------------
CWP2 SKTASTSRSRTAPKKTVQSRSSVTGNASRSAVARPTARAITKPTQSKRAPVRALPRSTNK 356
CWP3 ------------------------------------------------------------
                                                ΔPS3

CWP1 ------
CWP2 PIVRRR 362
CWP3 ------
```
