# Supplementary figures and images for: Selective Condensation Drives Partitioning and Sequential Secretion of Cyst Wall Proteins in Differentiating Giardia lamblia
Source: PLoS Pathog. 2010 Apr 8;6(4):e1000835. doi: 10.1371/journal.ppat.1000835 (PMC2851657; doi:10.1371/journal.ppat.1000835)

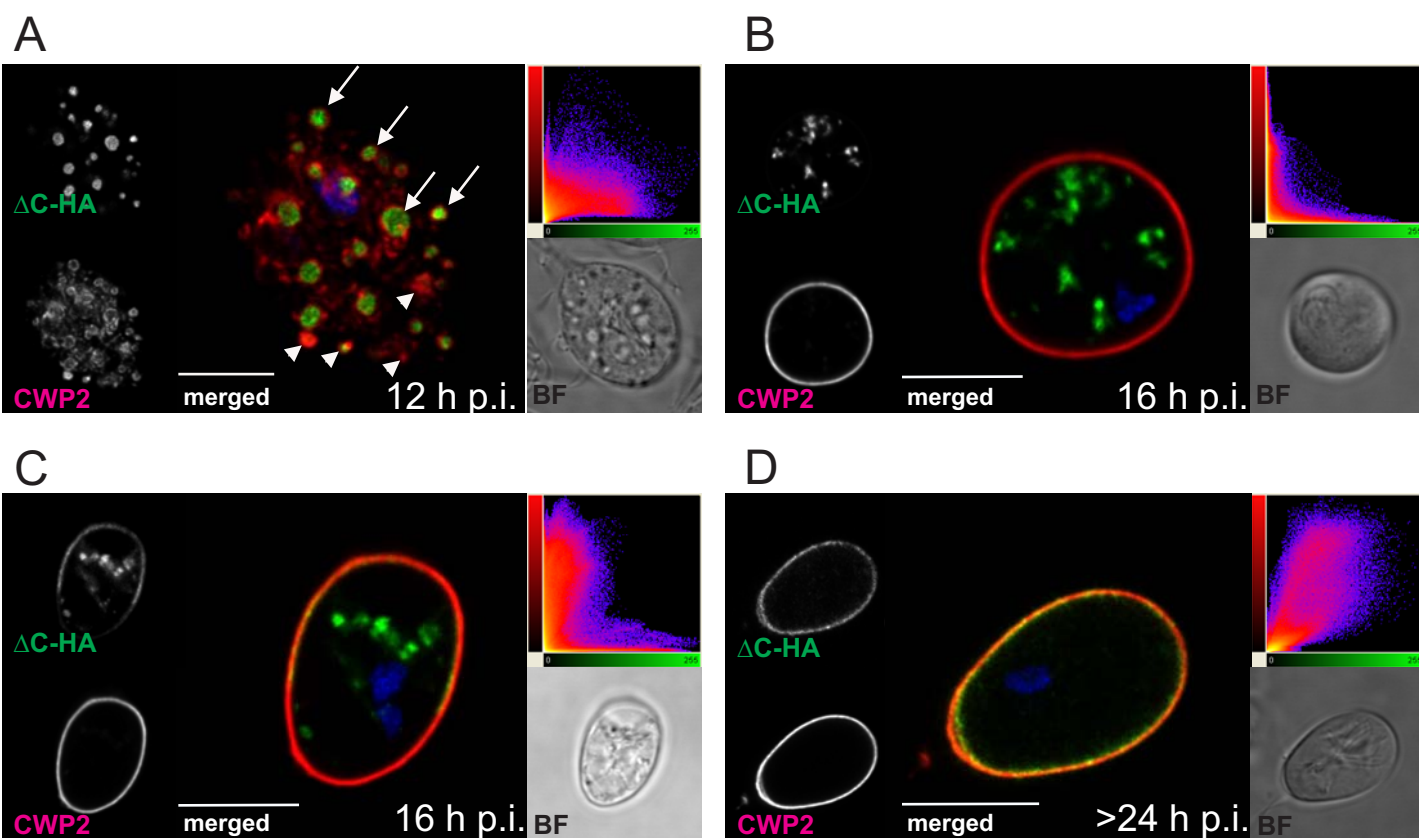

Supplement: Figure S2 — Localization of endogenous CWP2. Giardial CWP2 (red) and ΔC-HA (green) in a representative encysting cell (A) and in maturing cysts (B–D). An anti-CWP2 mAb was used to confirm the localization of the N-terminal processing product of CWP2 in differentiating cells between 12 h and >24 h p.i. Cells at 12 h p.i. show clear evidence for cargo partitioning (arrows) and sorting (arrowheads). Scale bar 5 µm. (0.20 MB PDF) [file ppat.1000835.s003.pdf]

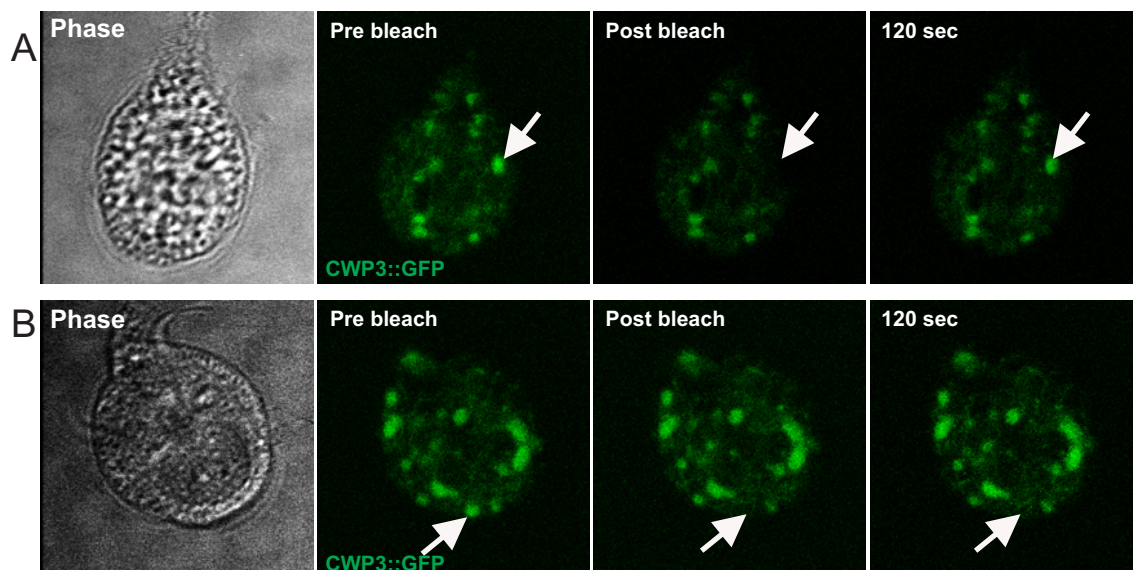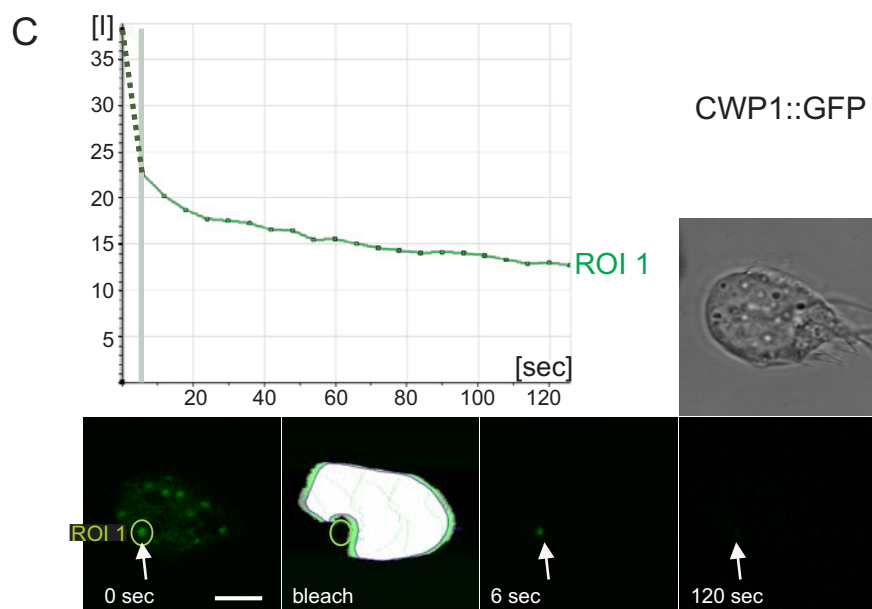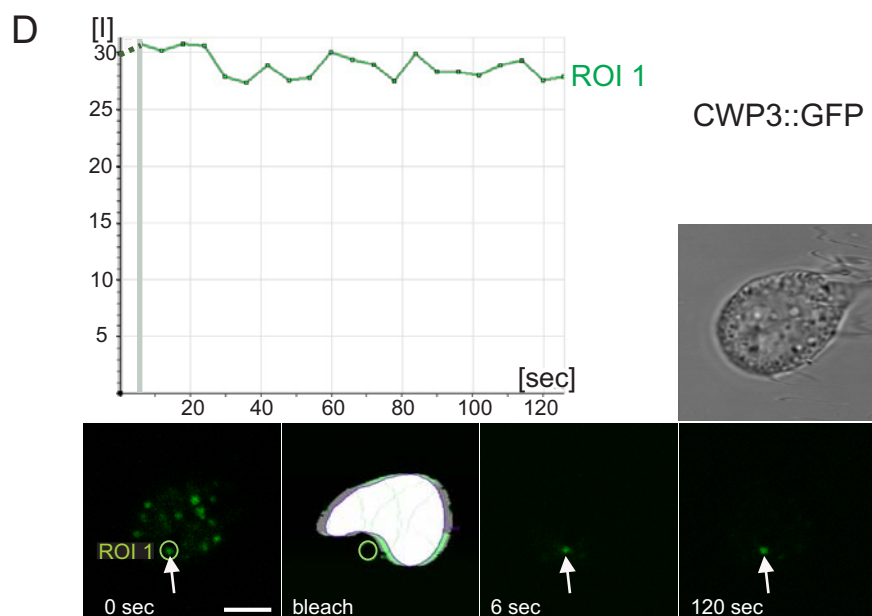

Supplement: Figure S3 — FRAP and FLIP analysis. Single frames from the FRAP analysis of the CWP3::GFP reporter (A, B). Pre bleach, post bleach and endpoint (120 sec) of the FRAP time-lapse series (28 images each) shown in Figure 3. First Row (A) 6 h p.i., second row (B) 12 h p.i. Arrows point to bleached organelle. Fluorescence loss in photobleaching (FLIP) experiments with CWP1::GFP (C) and CWP3::GFP (D) in cells at 12 h p.i. All GFP fluorescence in ESVs except in a single target organelle (ROI 1, arrow) was photobleached with 6 rapid cycles. The mobility of the remaining fluorescent reporter was quantified over 120 sec (graphs). Almost complete loss of CWP1::GFP fluorescence in the target organelle is consistent with mobility of the reporter in the ESV system. Conversely, the CWP3::GFP signal remains intact, consistent with immobilization due to incorporation into a condensed structure within ESVs. (0.43 MB PDF) [file ppat.1000835.s004.pdf]

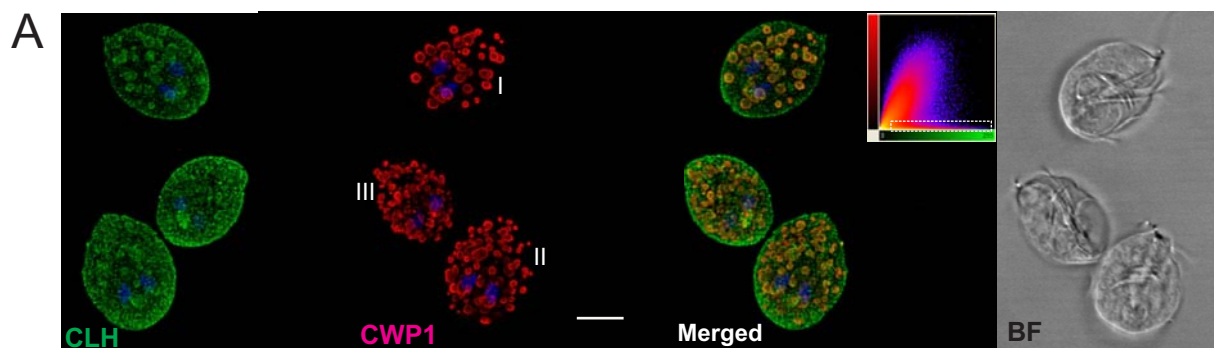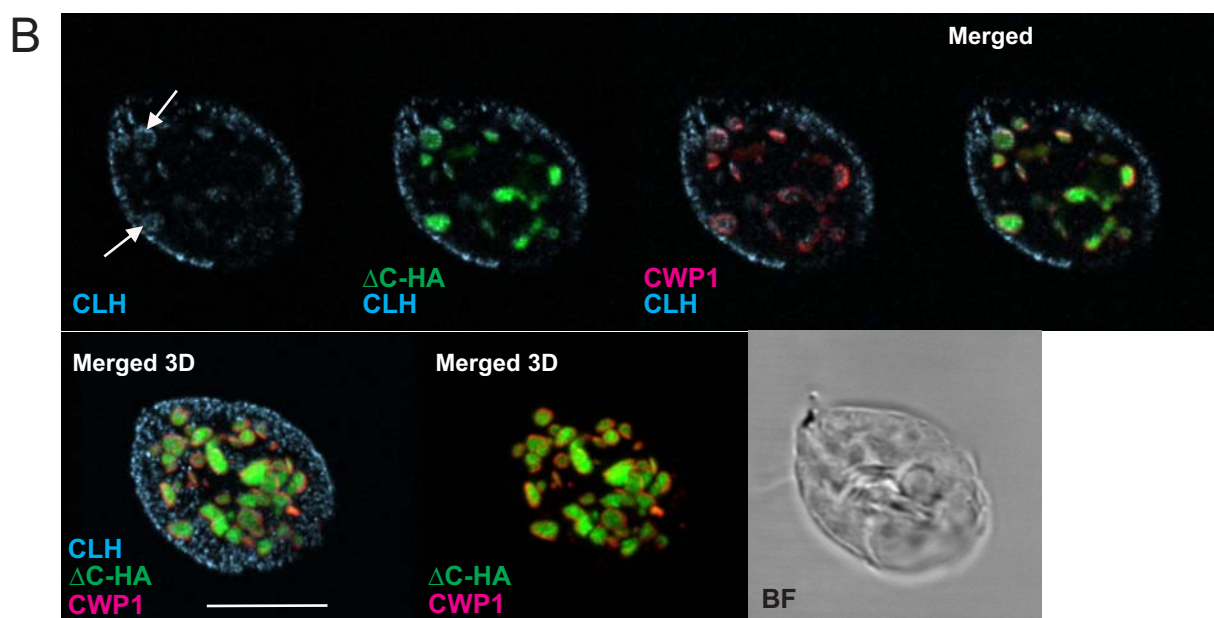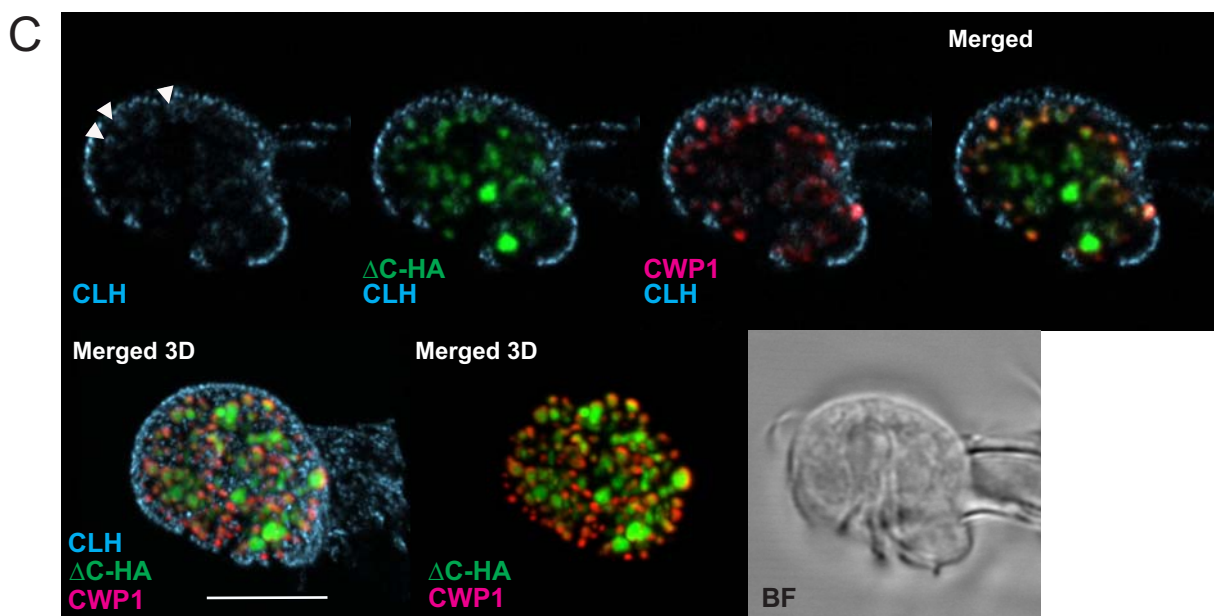

Supplement: Figure S4 — High resolution confocal microscopy of clathrin and secreted CWM. Clathrin heavy chain (CLH) is recruited to ESVs in cells showing evidence for cargo partitioning. CLH (green or blue) is detected by a specific antibody against the giardial protein [31] and the anti-CWP1 mAb (red) and/or anti-HA (green) is used to localize CWMfl and CWMco, respectively. Dual labeling (A) of encysting cells at 12 h p.i. showing full cargo partitioning (I) and beginning sorting (II and III) of CWP1. Recruitment of CLH to ESVs is most pronounced before and also during sorting. Three dimensional reconstructions of deconvolved optical sections and quantification of colocalization is shown (scatter plot; signal in boxed area represents CLH localized to peripheral vesicles). Triple labeling (B and C) of cells at 12 h p.i. showing full cargo partitioning (B) and sorting (C) of CWP1 (red) and ΔC-HA (green). CLH (blue) distributes to endosomal-lysosomal peripheral vesicles as shown previously [31], and is recruited to ESV membranes (arrows and arrowheads). Single optical sections (top rows) and three dimensional reconstructions (bottom rows) are shown. Scale bars 5 µm. (0.20 MB PDF) [file ppat.1000835.s005.pdf]

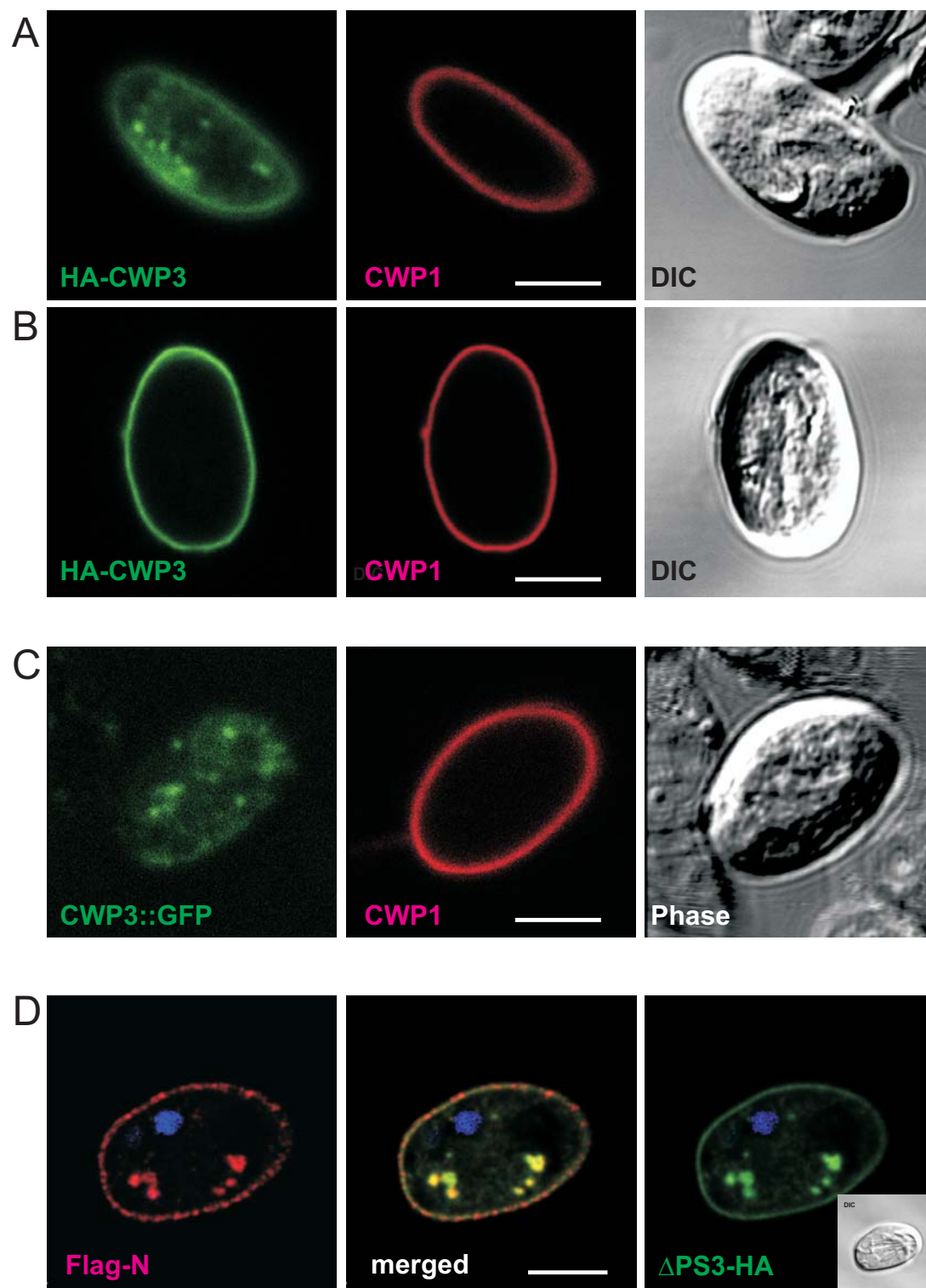

Supplement: Figure S5 — Localization of tagged CWP3 variants in cysts. Confocal fluorescence microscopy images of HA-CWP3 at early (A) and late (B) stages of cyst maturation. CWP3::GFP shows identical distributions in maturing live cysts (C). Scale bar 5 µm. (D) Localization of the uncleavable ΔPS3 variant of CWP2 shows the typical distribution of material exported with the CWMco fraction in maturing cysts. Scale bar 5 µm. (0.09 MB PDF) [file ppat.1000835.s006.pdf]
